# The psychological impact of the COVID-19 pandemic on medical students in Turkey

**DOI:** 10.12669/pjms.36.6.2985

**Published:** 2020

**Authors:** Fuat Torun, Sebahat Dilek Torun

**Affiliations:** 1 Prof. Dr. Fuat Torun, Department of Psychiatry, Istanbul Yeni Yuzyil University Medical Faculty, Istanbul, Turkey; 2 Dr. Sebahat Dilek Torun, Associate Professor, Department of Public Health, Bahcesehir University Medical Faculty, Istanbul, Turkey

**Keywords:** Novel coronavirus, Medical Students, Perceived stress, Impact of events, Anxiety

## Abstract

**Objective:**

We aimed to investigate the knowledge of medical students about COVID-19, the effects of the traumatic situation they experienced, the stress they perceived and the factors affecting them. In addition, we aimed to learn the thoughts of the students about the virus due to the uncertainties.

**Methods:**

The study was carried out online between April 30, May 5, 2020 with a questionnaire prepared with googleforms. For the study, all students studying at the Faculty of Medicine of Istanbul Yeni Yüzyıl University were called through class representatives and WhatsApp class groups. The questionnaire included sociodemographic information, knowledge and sources of information about the disease, to agreement degree the proposition whether covid 19 is produced as a biological weapon. The Perceived Stress Scale (PSS) and Impact of Events Scale-Revised (IES-R) were applied.

**Results:**

The total number of participants was 275 students. No student was infected with COVID-19 at the time of the survey. The presence of chronic disease in the participants was found to be a factor that increased anxiety (p = 0.01). Majority of participants (60.40%) stated that they agree with COVID-19 is a biological weapon. The mean scores of women ‘s total PSS and IES-R were higher than men. It was found that the families of the students had a lower monthly income than the minimum monthly wage is increasing the anxiety about getting COVID-19 infection and perceived stress. One-third of the students reported that sleep and appetite were impaired than the before pandemic. The announcements and website of Ministry of Health and the social media was the main source of information of the participants.

**Conclusions:**

It was found that medical students were highly worried about being infected with COVID-19. The scores obtained from the pre-clinic students’ anxiety to become infected with COVID-19, PSS and IES-R total scores were found to be significantly higher than their clinical students.

## INTRODUCTION

Due to the high rate of spread and mortality of COVID-19, majority of the society are experiencing anxiety in the face of increasing number of deaths all over the world. Recent research on COVID-19 reveals that healthcare workers (HCWs) experience high levels of stressful and traumatic events, and that their mental health is negatively affected including stress-related symptoms, depression, anxety and insomnia.^1^

Studies conducted on medical students during the epidemic show that they experience high level of anxiety because medical students are more likely to encounter with COVID-19 infected individuals and do not have sufficient information.^2^ The lack of adequate knowledge of medical students can cause exaggeration of the situation and can cause increase in stress and anxiety.^3^

In this study, we aimed to investigate the knowledge of medical students about COVID-19, the effects of the traumatic situation they experienced, the stress they perceived and the factors affecting them. This study also investigated the effect on these variables of potentially affecting factors such as age, gender, marital status, place of residence, accompanying chronic disease, a COVID-19 friend or relative, living with high risk persons such as someone aged above 65, pregnant woman and healthcare workers. In addition, we aimed to learn the thoughts of the students about the virus due to the uncertainties.

## METHODS

At the end of April, when the data were collected, universities were still closed, distance education was underway and a curfew was imposed under the age of 20.

### Study design and participants

For the study, all students studying at the Faculty of Medicine of Istanbul Yeni Yüzyıl University were called through class representatives and WhatsApp class groups. The faculty has a total of 433 students. The number of students completing the questionnaire was 275. Istanbul Yeni Yuzyil University Science Social and Noninterventional Health Sciences Research Ethical Comittee was aproved the study (Number: 2020 / 04-05).

### Survey Instruments

The study was carried out online between April 30, May 5, 2020 with a questionnaire prepared with Google Forms. In the initial part of the survey, information about the study was given and electronic approvals of the participants were obtained. In the first part of the questionnaire, the questions about the sociodemographic information of the participants prepared by the researchers are included. In the second part, where the participants live and the chronic disease of the people they live with, healthcare workers, pregnant, elderly above 65 years old, sources of information about COVID-19, whether someone who has had a COVID-19 infection or died recently. The survey included a question about participation in the proposition “COVID-19 is a biological weapon that was produced by human beings at the laboratories.”

To asses anxiety of being infected with COVID-19 we used a single 10 pointed ordinal visual scale, ranging from 0 ‘not at all anxious’ on the left-hand side to 10 ‘extremely anxious’ on the right–hand side.

### Perceived Stress Scale

Emotional stress of COVID-19 was evaluated using a Perceived Stress Scale (PSS) translated into Turkish. The PSS consisted of 14 items scored between 0 (never) and 4 (very often). Higher scores indicate greater perceived stress.^4^

### Impact of Events Scale-Revised

The psychological impact of COVID-19 was measured using the Impact of Event Scale-Revised (IES-R). IES-R measures PTSD symptoms in survivorship after an event. The respondent is asked to rate how much he or she has bothered by each symptom over the past week on a 5-point scale ranging from 0 to 4.^5^

### Statistical Analysis

Basic descriptive statistics were computed for all variables and reported as number of cases (frequency) and percentage for categorical variables and means and standart deviation (SD) or medians as appropriate for continuous variables. Association of presence of anxiety and categorical variables is analysed with the Chi-square (χ^2^) test and while students t-test and one-way analysis of variance (ANOVA) is used for continuous variables. Statistical Package for Social Sciences (SPSS) version 25.0 was used for analyses. All significance test were two-tailed and p<0.05 is accepted for statistical significance.

## RESULTS

About 60.7% of the participants (n = 167) were female, 39.3% (n = 108) were male and the mean age was 22.10 ± 2.69. No student was infected with COVID-19 at the time of the survey. The responses of the participants to the question of whether they are worries about being infected with COVID-19 and the factors affecting are given in Table-I.

**Table-I:** Worry about infection, perceived stress and impact of events scale and related factors.

		Anxiety		PSS Total		IES-R Total	

	N(%)	Mean(SD)	p	Mean(SD)	p	Mean(SD)	p
***Does the risky group live at home?***							
Yes	172(62.5)	6.00(2.44)	0.813	33.16(7.83)	0.621	36.54(17.66)	0.984
No	103(37.5)	5.92(2.74)		33.63(7.58)		36.58(16.33)	
***Gender***							
Male	108(39.3)	5.62(2.74)	0.07	31.19(7.44)	0.00	33.71(17.04)	0.02
Female	167(60.7)	6.20(2.40)		34.72(7.61)		38.40(17.01)	
***Marital Status***							
Single- No emotional relationship	173(62.9)	5.92(2.43)	0.90	33.05(7.08)	0.26	35.71(17.46)	0.00
Single- Has emotional relationship	97(35.3)	6.06(2.66)		34.06(8.71)		39.13(16.04)	
Married	5(1.8)	6.00(4.63)		29.00(8.39)		16(10.81)	
***Having chronic Disease***							
No	232(84.4)	5.81(2.52)	0.01	33.26(7.45)	0.74	36.43(17.25)	0.76
Yes	43(15.6)	6.84(2.57)		33.74(9.13)		37.26(16.74)	
***Residence***							
With families	214(77.8)	5.93(2.50)	0.63	33.09(7.87)	0.31	35.16(17.09)	0.01
Other	61(22.2)	6.11(2.74)		34.18(7.18)		41.46(16.53)	
Family’s monthly income*							
Below minimum mothly wage	27(9.8)	7.19(2.23)	0.00	36.59(6.86)	0.03	44.89(17.25)	0.06
2xminimum mothly wage	61(22.2)	6.33(2.61)		32.74(7.07)		38.25(17.73)	
3xminimum mothly wage	76(27.6)	6.14(2.45)		33.76(8.19)		35.08(16.95)	
4xminimum mothly wage	52(18.9)	5.38(2.57)		31.02(6.87)		34.31(15.88)	
Above 5xminimum mothly wage	59(21.5)	5.34(2.52)		33.95(8.37)		34.88(17.14)	
***Knowledge prevention/transmission***							
Yes	210(76.4)	5.79(2.57)	0.02	32.35(7.52)	0.00	34.67(16.78)	0.00
No/Not sure	65(23.6)	6.57(2.40)		36.52(7.56)		42.66(16.99)	
Biological weapon**							
Agree	166(60.4)	6.23(2.42)	0.02	33.16(7.62)	0.89	36.30(17.67)	0.95
Disagree	74(26.9)	5.28(2.60)		33.05(7.85)		36.99(16.59)	
No idea	35(12.7)	6.20(2.84)		33.71(8.15)		36.89(15.66)	
***Infected relatives/family members***							
Yes	89(32.4)	6.30(2.70)	0.14	33.17(8.97)	0.82	40.30(17.19)	0.01
No	186(67.6)	5.81(2.47)		33.41(7.07)		34.76(16.87)	
***Died relatives/family members***							
Yes	45(16.4)	6.38(2.81)	0.28	34.75(7.37)	0.24	38.40(17.78)	0.44
No	230(83.6)	5.89(2.50)		31.08(7.78)		36.20(17.03)	
Class							
Preclinic (1,2,3)	169(61.5)	6.21(2.52)	0.00	34.15(6.86)	0.00	38.82(16.76)	0.00
Clinic (4,5,6)	106(19.3)	5.59(2.57)		35.08(7.88)		32.94(17.20)	

SD: Standart deviation, Anxiety: anxiety of being infected with COVID-19,

10 pointed visual ordinal scale. PSS: Perceived Stress Scale, IES-R: Impacts of Events Scale-Revised.

* Monthly income: The self-reported monthly income of the family.

** Biological weapon: participation in the proposition that the virus was produced as a biological weapon.

Accompanying chronic disease was found to be a factor that increased anxiety (p = 0.01). The family’s income of students below the minimum monthly wage increases anxiety (p = 0.00). A significant difference was found in terms of anxiety between knowing the ways of transmission and prevention of COVID-19 infection and not (p = 0.02). The participants were asked whether they participated in the proposition to “COVID-19 was produced in the laboratories as a biological weapon”. 60.40% (n = 166) of the participants stated that they agree with this proposition. While this rate is 67.45% (n = 114) in the preclinic classes, this rate is 49.05% (n = 52) in the classes where there are clinical internships and the differences between groups were statistically significant (p=0.02). Those who do not believe that “COVID-19 was produced as a biological weapon” were found to be less worried about infected with COVID-19 (p = 0.02).

The responses of the participants to the PSS and affecting factors are given in Table-I. The perceived stress level in women was higher than in men (p = 0.00). The family’s income below the minimum monthly wage was found to higher level of the the perceived stress (p = 0.03). The mean perceived stress score was higher among the participants who stated that they don’t know the way of transmission and protection from COVID-19. Percevied Stress Scale scores were higher among clinical phase students compared to preclinical phase students (p=0.00).

The responses of respondents to the IES-R and affecting factors are given in Table-I. The average scores given to IES-R in women were also higher (p = 0.02). The average scores of married people are lower (p = 0.00). At the time of the survey, IES-R scores of those living with their family were significantly lower than the others (p = 0.01). A significant difference was found in terms of IES-R scores between knowing the transmission and prevention of COVID-19 infection and not (p = 0.00). Having a friend or relatives infected with COVID-19 caused a significant increase in IES-R scores (p = 0.01). A significant difference was found between the students’ classes in terms of IES-R scores (p = 0.00).

After the COVID-19 pandemic, the sleep patterns of the participants changed as 29.5% (n = 81) asleep late, wake up frequently and cannot fall asleep. 36.4% (n = 100) of the participants stated that their appetite after COVID-19 was worse than before and they started eating irregularly. The main resources used by the participants to be informed about COVID-19 was also asked. 56.4% of the participants stated that they used the official statements of the Ministry of Health and 43.6% always used social media as a source of information.

No significant difference was found in terms of whether smoking or not, number of rooms and living people under the risk such as older than 65 years, being pregnant and HCWs on worried about being infected with COVID-19, PSS and IES-R total scores (P> 0.05).

## DISCUSSION

In our study, the mean scores of females total PSS and IES-R were higher than men. In another study conducted with university students during the COVID-19 pandemic, it was reported that there was no gender-related differences between male and female students in terms of stress and negative affect.^2^

Some groups are at risk for COVID-19 infection.^6^-^8^ In our study, the students living the place where they were staying with who are in the risk group such as at the age of over 65 years , as pregnant and HCWs, did not make a significant difference in anxiety of being infected with COVID-19, PSS and IES-R total scores.

In our study, the presence of chronic disease in students was found to be a factor that increased anxiety of being infected with COVID-19. In a study, the presence of a chronic disease was found to be associated with elevation in IES-R, depression and anxiety scores.^9^ Living with the family makes a significant difference in IES-R total scores, while anxiety about infected with COVID-19, PSS total scores did not. In our study we found that having a friend or relatives infected with COVID-19 caused a significant increase in IES-R scores. In another study conducted during this period infection of one of the family or relatives with COVID-19 caused a significant increase in the IES-R total scores.^2^

We also found that the families of the students had a lower monthly income than the minimum monthly wage is increasing the anxiety about getting COVID-19 infection and perceived stress. Another study found that low family monthly income increased perception of danger and higher anxiety level. This situation was explained by the families of low income groups who need daily income and experience financial problems in the quarantine period.^10^

Despite increasing scientific publications in the literature on COVID-19, some aspects of the disease have not been clearly identified yet. This uncertainty causes an increase in misinformation and conspiracy theories about viruses and diseases.^11^ In this study, we found that 60.4 % of medical students who participated in this survey believes that COVID-19 was produced as a biological weapon in the laboratories. In our study, those who did not believe that COVID-19 was produced as a biological weapon in the laboratories were found to be less anxious about being infected with COVID-19. The low level of knowledge of students about COVID-19 symptoms significantly increased their participation in the proposition that the virus was produced as a biological weapon. Similar results were found in a study among university students regarding COVID-19.^10^

In Turkey the first three years of the medical school are theoretical courses and the last three years are clinical internships. The scores obtained from the pre-clinic students’ anxiety to become infected with COVID-19, PSS and IES-R total scores were found to be significantly higher than their clinical students. This result was attributed to the low level of knowledge of students in preclinical classes compared to clinical students.

A significant number of participants stated that they followed the information and developments about COVID-19 from the official statements of the Ministry of Health. Almost half of the participants also stated that they always use social media as a source of information. Smiliar results were found in another studies. 6,12

### Limitations in our study

Our research was done only in one medical school. For this reason, it does not represent all medical faculties. Participation in the research was voluntary. For this reason, some students did not complete the questionnaire although it was filled by the majority of students. The qualifications of those who did not complete the questionnaire are not fully known. We think that this study is important for understanding the stress experienced by medical students. In the light of this study, the data to be obtained as a result of new studies with larger series will be important.

## CONCLUSION

In our study, it was found that medical students were highly worried about being infected with COVID-19. In this study, the mean scores of women’s total PSS and IES-R were higher than men. Low montly income level of the family was an important factor that increases stress. Students studying in preclinical classes have less knowledge than clinical internship students. A significant part of the participants agreed that the COVID-19 was produced in the laboratories as a biological weapon. It has been determined that participation in this proposition is related to the low level of knowledge about COVID-19. The source of the participants’ basic information about COVID-19 was determined as the Ministry of Health’s announcements and the website and social media.

### Author’s Contribution:

**FT & SDT:** conceived the idea.

**FT:** collected the data.

**FT & SDT:** wrote the first draft.

**FT:** revised and translated the manuscript.

All authors reviewed and participated in preparation of the final version of the manuscript.
